# RNF115 aggravates tumor progression through regulation of CDK10 degradation in thyroid carcinoma

**DOI:** 10.1007/s10565-024-09845-w

**Published:** 2024-02-20

**Authors:** Jinxiang Zhu, Longwei Guo, Hao Dai, Zhiwei Zheng, Jinfeng Yan, Junsong Liu, Shaoqiang Zhang, Xiang Li, Xin Sun, Qian Zhao, Chongwen Xu

**Affiliations:** 1 Department of Otorhinolaryngology-Head and Neck Surgery, the First Affiliated Hospital of Xi’an Jiaotong University, Western Yanta Road, Xi’an City, 710061 Shaanxi Province China; 2 Department of General Surgery, Shaanxi Provincial Cancer Hospital, Xi’an City, 710061 Shaanxi Province China; 3 Department of Radiation Oncology, the First Affiliated Hospital of Xi’an Jiaotong University, Xi’an City, 710061 Shaanxi Province China; 4 Department of The Third Ward of General Surgery, Rizhao People’s Hospital, Rizhao City, 276800 Shandong Province China; 5 Department of Thoracic Surgery, the First Affiliated Hospital of Xi’an Jiaotong University, Xi’an City, 710061 Shaanxi Province China

**Keywords:** Thyroid carcinoma, RING finger protein 115, Cyclin-dependent kinase 10, Ubiquitination, Raf-1 pathway, Cell cycle

## Abstract

**Background:**

RING Finger Protein 115 (RNF115), a notable E3 ligase, is known to modulate tumorigenesis and metastasis. In our investigation, we endeavor to unravel the putative function and inherent mechanism through which RNF115 influences the evolution of thyroid carcinoma (THCA).

**Methods:**

We analyzed RNF115 expression in THCA using the Cancer Genome Atlas (TCGA) database. The influence of RNF115 on the progression of THCA was evaluated using both *in vitro* and *in vivo* experimental approaches. The protein regulated by RNF115 was identified through bioinformatics analysis, and its biological significance was further explored.

**Results:**

In both THCA tissues and cells, RNF115 showed elevated expression levels. Enhanced expression of RNF115 fostered cell proliferation, tumor growth, and the exacerbation of epithelial-mesenchymal transition (EMT) in THCA, while also promoting tumor lung metastasis. Bioinformatics analysis identified cyclin-dependent kinase 10 (CDK10) as a downstream target of RNF115, which was found to be ubiquitinated and degraded by RNF115 in THCA cells. Functionally, overexpression of CDK10 was found to counteract the promotion of malignant phenotype in THCA induced by RNF115. From a mechanistic perspective, RNF115 activated the Raf-1 pathway and enhanced cancer cell cycle progression by degrading CDK10 in THCA cells.

**Conclusion:**

RNF115 triggers cell proliferation, EMT, and tumor metastasis by ubiquitinating and degrading CDK10. The regulation of the Raf-1 pathway and cell cycle progression in THCA may be profoundly influenced by this process.

**Supplementary Information:**

The online version contains supplementary material available at https://doi.org/10.1007/s10565-024-09845-w.

## Introduction

Thyroid carcinoma (THCA) is the predominant malignancy of the endocrine system, constituting approximately 95% of all endocrine tumors (Haroon Al Rasheed and Xu 2019; Hu et al. 2021). In 2020, the estimated incidence of THCA in the United States stood at 52,890 cases, with anticipated fatalities accounting for approximately 4.1% (2180 patients) (Hu et al. 2021). Moreover, the incidence of THCA is exhibiting a steady upward trend, increasing approximately 4% annually (Hu et al. 2021). The oncogenesis and progression of THCA are regulated by oncogenic and tumor-suppressor genes and pathways. The identification of novel biomarkers contributing to the initiation and advancement of THCA could potentially enhance the development of targeted therapeutics and diagnostic approaches for this condition.

Ubiquitination, playing a pivotal role in protein posttranslational modification and pathological progression, is fundamental to tumorigenesis (Yu et al. 2019). This process involves three crucial enzymes: ubiquitin-activating enzyme (E1), ubiquitin-conjugating enzyme (E2), and ubiquitin ligase (E3) (Toma-Fukai, Shimizu 2021). The transfer of ubiquitin from an E2 to the target protein is particularly facilitated by E3s, which play a pivotal role in substrate selection (Toma-Fukai and Shimizu 2021). The RING finger (RNF) protein family, a complex group of E3s, exerts critical roles in tumor initiation and progression (Senft et al. 2018). For instance, RNF128 downregulation induces cellular epithelial-mesenchymal transition (EMT) and stemness in melanoma via the ubiquitination of CD44/CTTN, thus representing a potential diagnostic and prognostic biomarker (Wei et al. 2019). RNF220 enhances the stemness and malignant progression of colon cancer cells via the regulation of the USP22-BMI1 axis (Yan et al. 2021). Thus, the identification of cancer-related RNF family members can advance our understanding of the molecular mechanisms underlying tumor progression and offer new prognostic markers and therapeutic strategies.

In our current research, we highlight the increased expression of RNF115 in THCA, drawing data from the Cancer Genome Atlas (TCGA) repository. Consequently, we embarked on exploring the biological roles of RNF115 in THCA through *in vitro* and *in vivo* experimentation. Moreover, we identified CDK10 as a downstream target of RNF115 in THCA through bioinformatics analysis. Our research indicates that RNF115 acts as an oncogenic driver in THCA. Delving deeper into the mechanisms of RNF115 might offer valuable insights for advancing diagnostic, prognostic, and treatment approaches for THCA.

## Methods

### Data collection and processing

The TCGA-THCA RNAseq data were obtained from the UCSC database in FPKM format. The UCSC Xena browser (http://xena.ucsc.edu/) was employed to evaluate RNF115 expression across various tumor types. This study included a total of 615 samples from three categories: normal solid tissues, primary tumor samples, and metastatic tumors. The GEPIA tool (http://gepia.cancer-pku.cn/) was used to examine RNF115 expression in paired and unpaired normal and tumor samples. The cutoff value for RNF115 expression was set at high (75%) and low (25%) in GEPIA. Log2(FPKM + 1) processing was applied to the corresponding numbers of adjacent and cancer samples. Visualization of the data was achieved using the ggplot2 package.

Unpaired sample plots were assessed via the Mann–Whitney U test, while paired sample plots were analyzed using a t-test.

### Samples

Tissue specimens were collected from THCA patients (n = 113) undergoing primary surgical resection at the First Affiliated Hospital of Xi'an Jiaotong University. Expression of RNF115 was analyzed in the collected tumor samples and their corresponding non-tumorous thyroid tissues. The procurement of tissue specimens received the endorsement of the Ethics Committee at the First Affiliated Hospital of Xi'an Jiaotong University (Approval no. 2021–1407). Informed consent for sample collection was acquired from each patient.

### Cell culture and treatment

Human thyroid regular cells (HTori-3) and THCA-specific cell strains (TPC-1, KTC-1, CAL62, and SNU-790) were sourced from Beina Biology (China). They were cultured in a DEME/F12 medium (Hyclone, UT, USA) supplemented with 10% fetal bovine serum (FBS) and a 1% antibiotic combination of penicillin/streptomycin, maintained at 37 °C in an atmosphere containing 5% CO_2_. TPC-1 and SNU-790 cells were transfected with vectors carrying RNF115 overexpression (Ad-RNF115), RNF115 knockdown (Ad-shRNA1 and Ad-shRNA2), negative controls (Ad-NC and Ad-shNC), and/or CDK10 overexpression, using the Lipofectamine™2000 transfection kit (Invitrogen, CA, USA). The designated sequences for shRNA include: shRNA-1: 5′-GCCGUGGCUAGAACUGCAUTT-3′ and shRNA-2: 5′-CGTCTGAATAGAATTAATT-3′. A fraction of the transfected cells was treated with cycloheximide (CHX, a protein synthesis inhibitor) for various durations (0, 4, 8, and 12 h). Meanwhile, another subset received a 10 μM dose of MG132, a proteasome inhibitor, for a duration of 8 h.

### Methylthiazoltetrazolium (MTT) assay

TPC-1 and SNU-790 cells, with a concentration of 1.5 × 10^4^/mL, were allocated into 96-well plates and then incubated for a period of 48 h. Post this incubation phase, 20 μL of 3-(4, 5-dimethylthiazol-2-yl)-2, 5-diphenyltetrazolium bromide was introduced to every well, followed by a further 4-h incubation period. Subsequently, the medium was aspirated, and the resulting formazan crystals were solubilized in 150 μL of DMSO per well. The viability of the cells was ascertained by gauging the absorbance at 490 nm with the aid of a spectrophotometer.

### Colony formation assay

The colony formation capacity of TPC-1 and SNU-790 cells was evaluated using a previously established method (Wei et al. 2019). Cells were dispensed into a 6-cm culture dish at a density of 1 × 10^3^ cells per dish. They were then allowed to grow for two weeks, with the culture medium being refreshed every three days. Subsequently, the cells were rinsed using phosphate-buffered saline (PBS), underwent fixation with a 4% paraformaldehyde solution, and were subsequently stained with a 0.4% crystal violet solution for a duration of 15 min. Following this, the colony count was determined.

###  in vivo tumor model

The First Affiliated Hospital Ethics Committee of Xi'an Jiaotong University granted approval for all procedures involving animals (Approval no. 2021–1407). Mice (sourced from Charles River, Beijing, China), aged 4–6 weeks, were randomly assigned to one of four groups: Ad-NC, Ad-RNF115, Ad-shNC, and Ad-shRNA1, with six mice in each group. TPC-1 cells that underwent stable transfection were administered into mice via the lateral tail vein injection. Tumor volume was documented at seven-day intervals. Thirty days post-injection, *in vivo* imaging of mice was conducted utilizing the IVIS Spectrum *In Vivo* Imaging System to monitor lung metastasis. Following imaging, mice were humanely euthanized, and tumor tissues were collected for further investigations. All animal procedures complied with the National Institutes of Health Laboratory Animal Care and Used Guidelines.

### Wound-healing assay

Cells that underwent transfection were placed in six-well culture plates and grown in a serum-deprived medium until they reached full confluence. Subsequently, a defined wound area was established by introducing a scratch into the cell monolayer using a 10-µL pipette tip. The state of the scratch wounds was documented at 0 and 24 h post-initiation through microscopic observation (Olympus, Japan).

### Transwell invasion assay

An invasion assay was executed utilizing 24-well transwell plates (Corning, NY, USA). Cells, with a concentration of 1 × 10^6^, were suspended in serum-free medium and introduced into the Matrigel-pre-coated upper chambers (BD Biosciences, NJ, USA). The bottom chamber was supplemented with 0.6 mL of DEME/F12 medium containing 10% FBS. After incubating for 24 h, cells were fixed with 4% paraformaldehyde and then stained with crystal violet. The number of cells that invaded was then quantified using a microscope.

### Immunofluorescence assay

An immunofluorescence assay was executed based on the methodology delineated by Sáenz et al. (2019). Initially, cells were fixed using 4% paraformaldehyde and permeabilized with 0.3% Triton X-100. After a blocking step with 5% FBS, the cells were exposed to primary antibodies, specifically anti-E-cadherin, anti-N-cadherin, anti-Flag, and anti-HA, and incubated at 4 °C overnight. Thereafter, they were subjected to secondary antibodies. To demarcate the nuclei, 4, 6-diamidino-2-phenylindole (DAPI; procured from Yeasen, China) was employed. Observations were made utilizing a confocal laser scanning microscope (Model LSM510; procured from Zeiss, Germany).

### Hematoxylin and eosin (HE) staining

Tumor tissue morphology was assessed via HE staining. Following the processes of deparaffinization and rehydration, tissue slices underwent staining procedures using hematoxylin and eosin dyes. The stained sections were subsequently examined under a microscope.

### Enrichment analysis

RNF115-associated genes were predicted via the BioGRID (https://thebiogrid.org/) and underwent enrichment analysis using Metascape (https://metascape.org/) and the R programming language. Enrichment analysis, incorporating both Gene Ontology (GO) and the Kyoto Encyclopedia of Genes and Genomes (KEGG), was conducted using a suite of ontology databases: KEGG Pathway, GO Biological Processes, Reactome Gene Sets, Canonical Pathways, CORUM, WikiPathways, and PANTHER pathways. Enriched terms were chosen based on the following criteria: a p-value below 0.01, an observed count of at least 3, and an enrichment factor greater than 1.5, with the enrichment factor delineating the ratio of observed counts relative to those anticipated by chance.

### Identification of critical genes interacted with RNF115.

The top 25 RNF115-interacting genes were identified via Pathway Commons (https://www.pathwaycommons.org/). Genes regulated by RNF115 were analyzed for their correlation with RNF115 in THCA. Data for THCA samples were obtained from the TCGA database via cBioPortal. We probed the expression of genes regulated by RNF115 via GEPIA, an online tool accessible at http://gepia.cancer-pku.cn/, leveraging data from the TCGA database.

### RT-qPCR

RNA from both tissues and cells was isolated utilizing the TRIzol reagent (Invitrogen, CA, USA). RT-qPCR was performed following a previously described protocol (Pan et al. 2019). PCR was performed using primers detailed in Supplementary Table S1.

### Western blotting

Protein extraction from tumor tissues and cells was performed using RIPA buffer, with the subsequent western blotting procedure carried out as detailed in earlier methods (Bacopulos et al. 2012). The primary antibodies used for western blotting were anti-RNF115 (0.4 µg/mL), anti-E-cadherin (1:10,000), anti-N-cadherin (1 µg/mL), anti-CDK10 (1:2,000), anti-SFN (2 µg/mL), anti-MYC (2 µg/mL), anti-p-Raf-1 (1:1,000), anti-Raf-1 (1:2,000), anti-p-MEK1/2 (1;1,000), anti-MEK1/2 (1:1,000), anti-p-ERK1/2 (1:1,000), anti-ERK1/2 (1 µg/mL), anti-CyclinD1 (1 µg/mL), anti-CDK4 (2 µg/mL), anti-Bax (1:100), anti-Cleaved caspase-3 (1:2,000), and anti-β-actin (1:5,000) (Invitrogen, CA, USA).

### Immunohistochemistry (IHC)

The IHC staining procedure was adopted. In a summarized protocol, paraffin-embedded tissue samples were sectioned into 4-μm slices. Following deparaffinization and rehydration, the sections underwent antigen retrieval using 1 mM EDTA (pH 8.0). To neutralize the tissue's inherent peroxidase activity, sections were treated with 0.3% hydrogen peroxide. Blocking was achieved using 5% goat serum. This was followed by an incubation phase with primary antibodies: anti-RNF115 (1:200), anti-CDK10 (1:200), anti-Ki67 (1:200), anti-E-cadherin (10 µg/mL), and anti-N-cadherin (1:100) sourced from Invitrogen, CA, USA. Subsequently, sections were incubated with biotinylated secondary antibodies and observed under a microscope (Olympus, Japan).

### Co-immunoprecipitation (CoIP)

Cells of the HEK-293 T line, numbered at 1 × 10^6^, underwent co-transfection utilizing constructs that harbored Flag-tagged RNF115 alongside HA-tagged CDK10. Following a 24-h period, cells were analyzed through western blot and immunohistochemistry (IHC) to assess the expression and localization of RNF115 and CDK10, utilizing primary antibodies against Flag and HA.

### Statistical analysis

*In vitro* studies were repeated on three separate occasions, whereas *in vivo* assays involved six biological repeats and three technical repetitions. Data analysis was executed using SPSS 27.0 software (IBM, IL, USA), with results expressed as the mean ± standard deviation. For comparisons involving multiple groups, one-way ANOVA was utilized. For pairwise comparisons, Tukey’s test was employed. A statistically significant result is indicated when *p* < 0.05.

## Results

### Upregulation of RNF115 in THCA tissues and cells

**Fig. 1:**
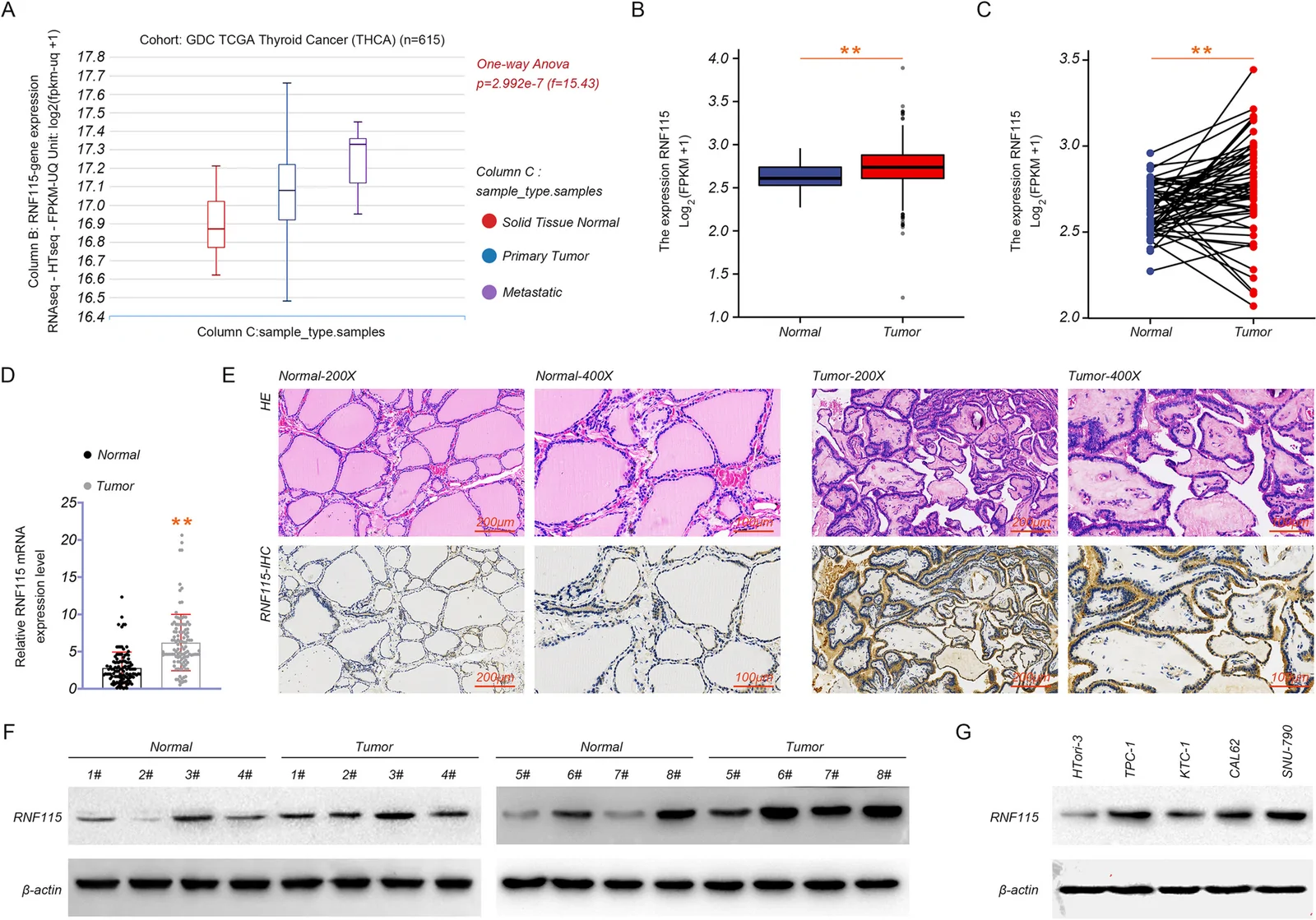
RNF115 expression is upregulated in thyroid carcinoma (THCA) tissues and cells. **A** The expression of RNF115 in normal solid tissues, primary tumors, and metastatic tumors of THCA is based on the TCGA database. **B-C** RNF115 expression in unpaired and paired tumor samples of THCA (**p < 0.01). **D-F** RNF115 expression in human normal and tumor tissues (n = 113) by RT-qPCR (**p < 0.01 *vs.* normal), HE staining, immunohistochemistry, and western blotting. **(G)** RNF115 expression in normal (HTori-3) and THCA cells (TPC-1, KTC-1, CAL62, and SNU-790) by western blotting

Our initial investigation centered on determining the expression levels of RNF115 in THCA tumor tissues and cell lines, aiming to shed light on its role in THCA progression. The UCSC analysis tool highlighted a distinct pattern of RNF115 expression derived from the TCGA-THCA database. Metastatic tumors displayed the highest expression, followed by primary tumors, and then normal solid tissues (*p* = 2.992e-7; Fig. 1A). This trend was further confirmed when examining THCA tumor tissues against normal tissues. Both paired and unpaired samples showcased elevated RNF115 expression in THCA tumor tissues (p < 0.01; Fig. 1B, C). Further evaluation of RNF115 expression in human THCA tumors and adjacent normal tissues (n = 113) revealed significantly elevated RNF115 levels in tumor tissues (*p* < 0.01; Fig. 1D-F). Analysis of correlations indicated that THCA patients with high RNF115 expression had larger tumor size (≥ 3 cm^3^) and advanced clinical stage (stage N1 and TNM stage III-IV) (Table 1). The histopathological type of THCA patients included follicular cancer, medullary thyroid carcinoma, and papillary cancer (Table 1). We found that RNF115 is localized in both nucleoli and mitochondria, predominantly in mitochondria (Fig. 1E). Expression levels of RNF115 were examined in human thyroid epithelial cells (HTori-3) and several THCA cell lines, including TPC-1, KTC-1, CAL62, and SNU-790. Our findings revealed that THCA cells exhibited notably elevated RNF115 expression in comparison to the normal HTori-3 cells, with TPC-1 and SNU-790 cells showing the most pronounced increase (Fig. 1G). Consequently, our subsequent research centered on these two cell lines, TPC-1 and SNU-790.

**Table 1:** Relationship between RNF115 expression in thyroid carcinoma and clinicopathological features of patients

Characteristics	Number	RNF115 Low expression (< medin)	RNF115High expression (≥ medin)	*P* value
Number	113	56	57	
Ages(years)				0.510
< 55	57	30	27	
≥ 55	56	26	30	
Gender				0.426
Female	83	43	40	
Male	30	13	17	
Tumor size(cm^3^)				0.001*
< 3	53	35	18	
≥ 3	60	21	39	
N-stage				0.001*
N0	39	32	15	
N1	74	24	42	
TNM stage				0.002*
I-II	46	27	19	
III-IV	67	29	38	
Histopathological type				0.772
Follicular cancer	36	19	17	
Medullary thyroid carcinoma	10	4	6	
Papillary cancer	67	33	34	

### RNF115 enhanced cell proliferation and tumor growth of THCA

**Fig. 2:**
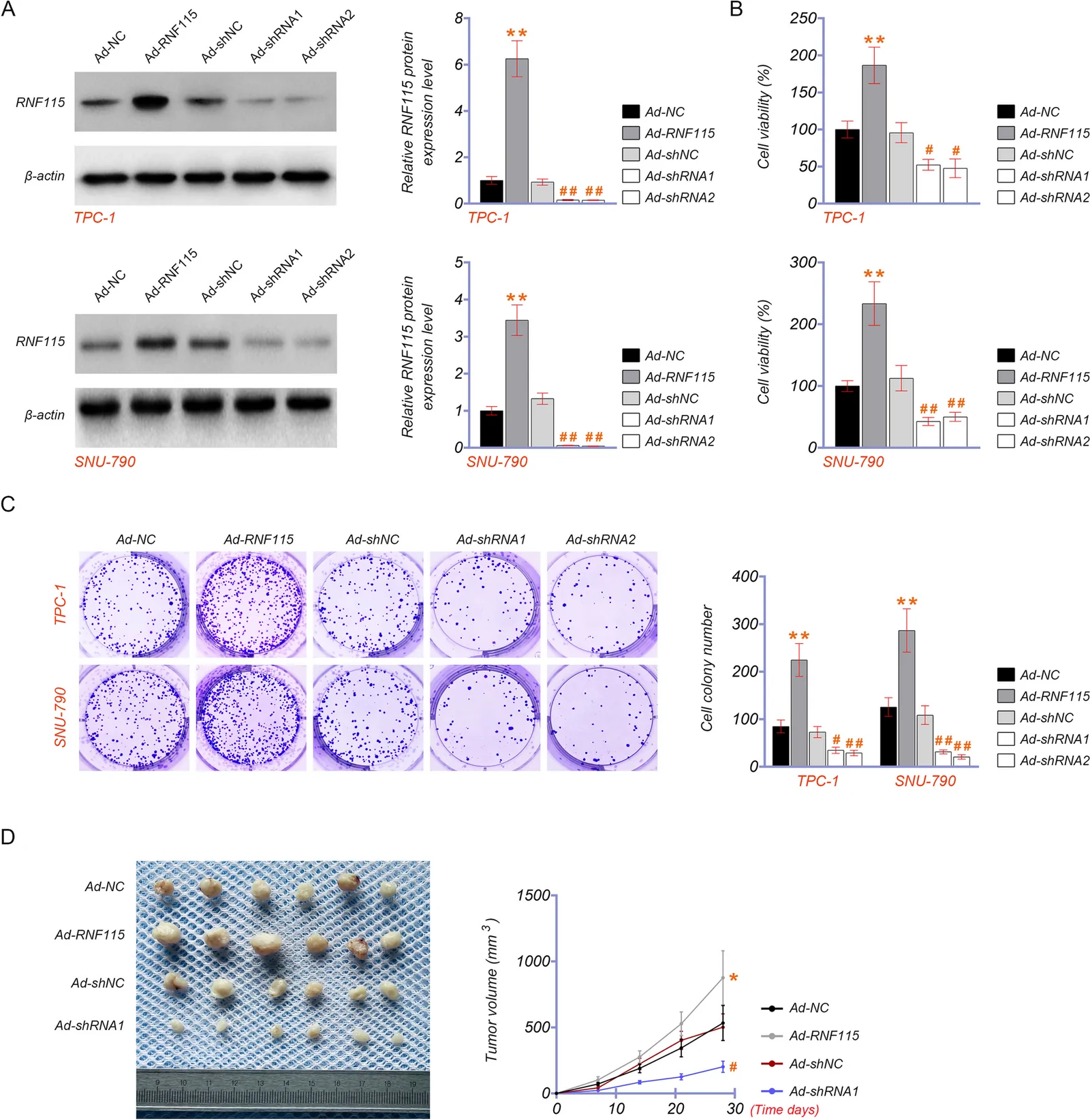
RNF115 promotes cell proliferation and tumor growth of thyroid carcinoma (THCA). **A** The protein expression of RNF115 in TPC-1 and SNU-790 cells. **B** Cell viability by MTT assay. **C** Cell proliferation by colony formation assay. **A-C** TPC-1 and SNU-790 cells were transfected with RNF115 overexpression (Ad-RNF115), knockdown (Ad-shRNA1 and Ad-shRNA2), or their negative controls (Ad-NC and Ad-shNC). **D** Xenograft Tumor volume. BALB/c mice were injected with stably transfected TPC-1 cells into the lateral tail vein. Tumor volume was recorded every seven days. *p < 0.05 and **p < 0.01 *vs.* Ad-NC; #p < 0.05 and ##p < 0.01 vs. Ad-shNC

To discern the function of RNF115 in THCA, we subjected TPC-1 and SNU-790 cells to RNF115 overexpression (Ad-RNF115) and knockdown (Ad-shRNA1, Ad-shRNA2). RNF115 expression in different groups was first assessed. Through western blot analysis, it was evident that the Ad-RNF115 group exhibited an upregulated RNF115 expression, while the Ad-shRNA1 and Ad-shRNA2 groups displayed diminished expression in comparison to the control group (*p* < 0.01; Fig. 2A). This underlines the successful accomplishment of the transfection process. Functionally, RNF115 overexpression bolstered TPC-1 and SNU-790 cell proliferation, while RNF115 knockdown hindered cell proliferation (*p* < 0.05; Fig. 2B, C). The role of RNF115 in THCA was further validated through *in vivo* experiments. BALB/c mice were transfected with either RNF115-overexpressed or -downregulated TPC-1 cells. The data showed that RNF115 overexpression augmented THCA tumor volume, while RNF115 knockdown impeded tumor growth (*p* < 0.05; Fig. 2D).

### RNF115 facilitates THCA metastasis in vitro and in vivo

**Fig. 3:**
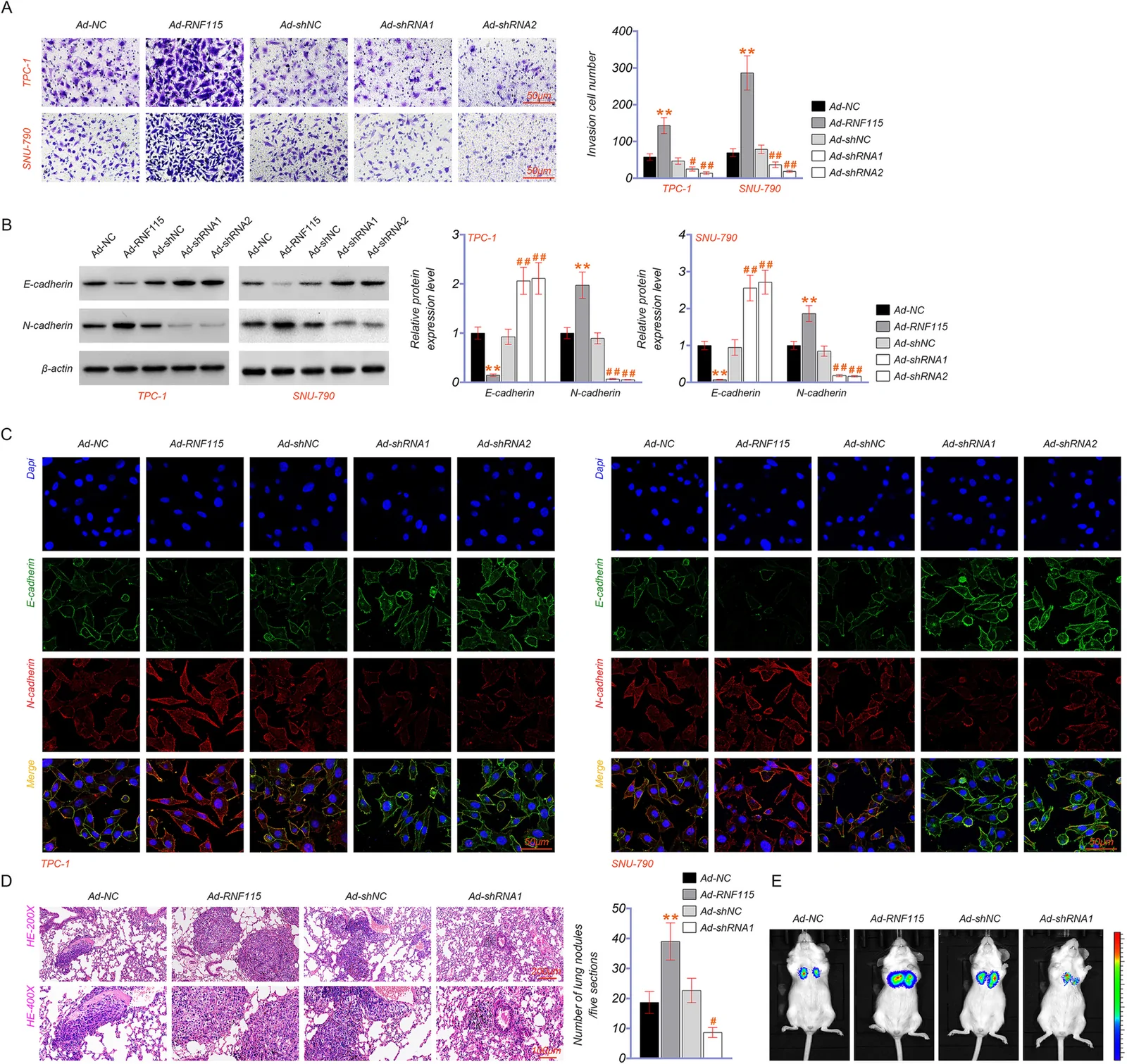
RNF115 promotes cell epithelial-mesenchymal transition (EMT) and tumor lung metastasis of thyroid carcinoma (THCA).** A** Cell invasion by Transwell assay (scale bar = 50 µm). **B-C** EMT-related proteins (E-cadherin and N-cadherin) were expressed in cells by western blotting and immunofluorescence (scale bar = 50 µm). **A-C** TPC-1 and SNU-790 cells were transfected with RNF115 overexpression (Ad-RNF115), knockdown (Ad-shRNA1 and Ad-shRNA2), or their negative controls (Ad-NC and Ad-shNC). **D-E** HE staining (200 × and 400 × magnifications) and imaging measured lung metastasis of the THCA tumor. BALB/c mice were injected with stably transfected TPC-1 cells into the lateral tail vein. Tumor volume was recorded every seven days. **p < 0.01 *vs.* Ad-NC; #p < 0.05 and ##p < 0.01 vs. Ad-shNC

Given that metastasis is a key malignant characteristic of THCA, we further investigated the effects of RNF115 on metastasis. *in vitro* experiments demonstrated that elevating RNF115 levels promoted the migratory and invasive capabilities of TPC-1 and SNU-790 cells. Conversely, inhibiting RNF115 produced the opposite effect (*p* < 0.05; Figure S1A & 3A). The Epithelial-mesenchymal transition (EMT) process, marked by a decline in E-cadherin and an increase in N-cadherin, underpins cellular metastasis (Shash et al. 2021; Mittal 2018). Our data indicated that RNF115 overexpression reduced E-cadherin levels and augmented N-cadherin expression in TPC-1 and SNU-790 cells. Conversely, RNF115 knockdown elicited the opposite effects (*p* < 0.01; Fig. 3B, C). *In vivo*, RNF115 overexpression significantly increased metastatic colonies in the lung tissues of mice transfected with TPC-1 cells, whereas RNF115 knockdown reduced metastasis (*p* < 0.01; Fig. 3D, E).

### Identification of key genes regulated by RNF115

**Fig. 4:**
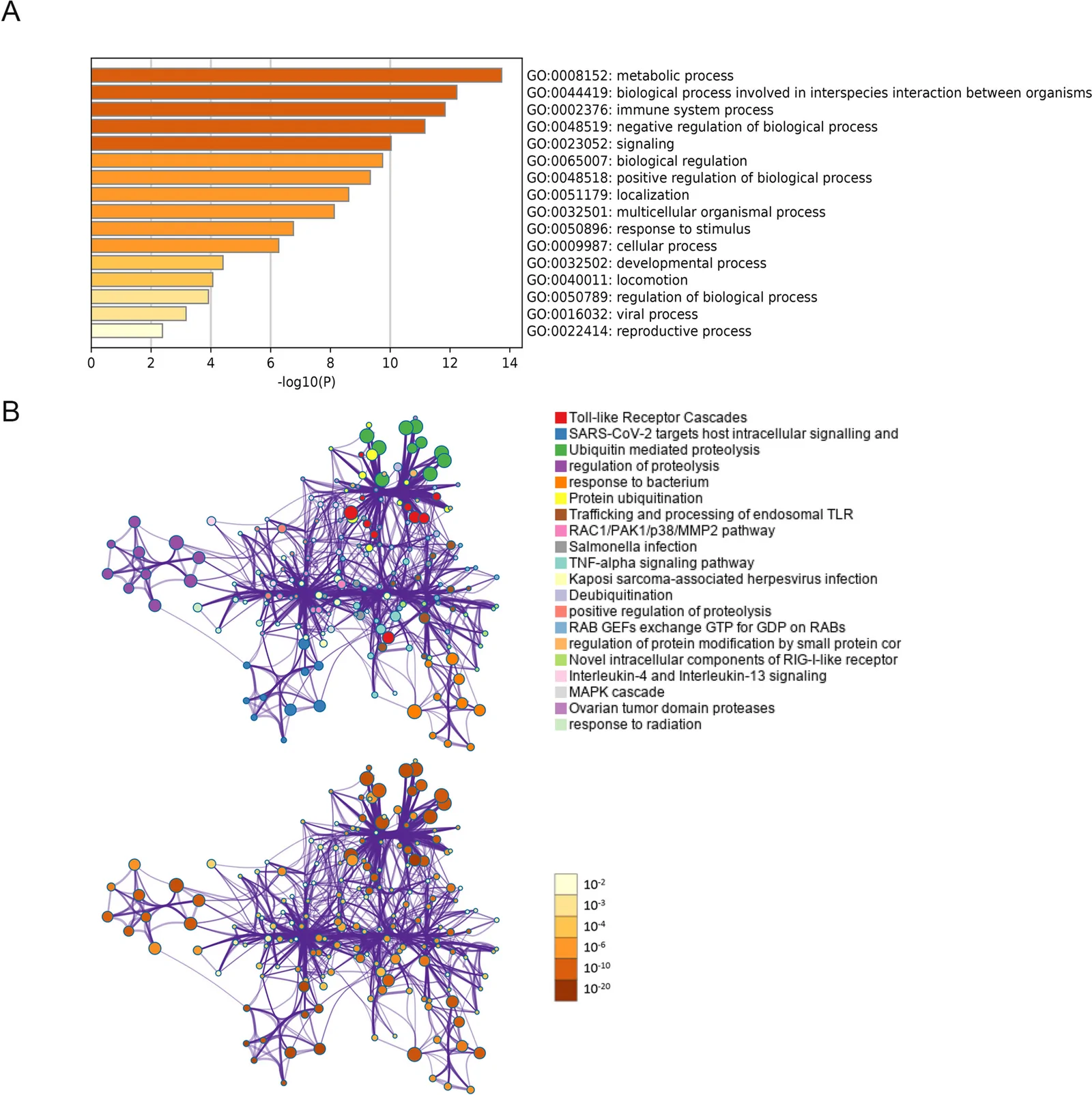
GO **(A)** and KEGG **(B)** enrichment analyses for genes interacting with RNF115

**Fig. 5:**
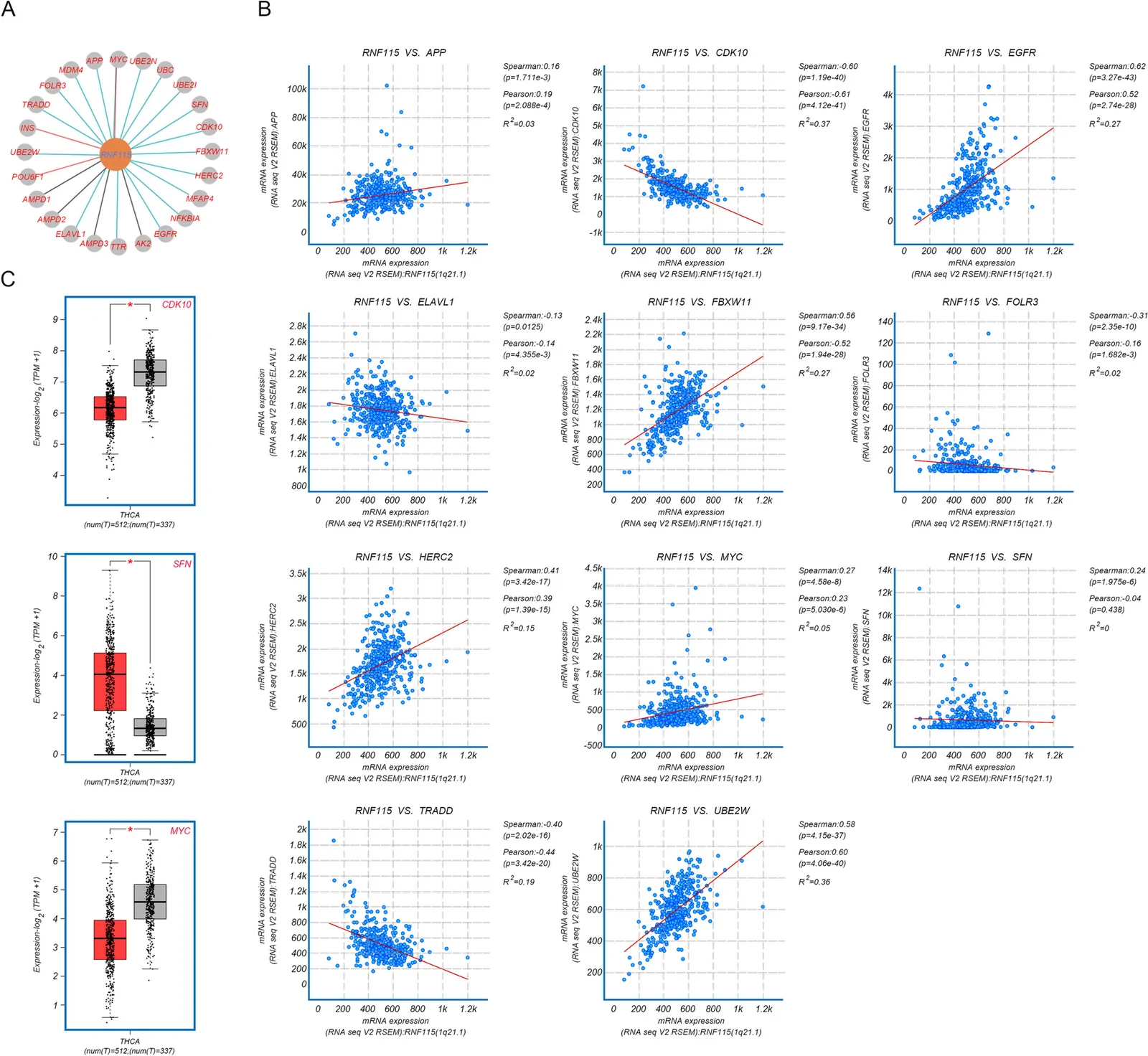
Identification of key genes regulated by RNF115. **A** Protein–protein interaction network for RNF115 and its interacted genes. **B** Correlation analysis for RNF115 and its interacted genes in thyroid carcinoma (THCA). **C** The CDK10, SFN, and MYC expression in average and THCA tumor samples were based on the TCGA database (*p < 0.05)

Genes interacting with RNF115 were predicted using BioGRID (https://thebiogrid.org/), followed by GO and KEGG enrichment analyses. As depicted in Fig. 4A, RNF115-interacting genes were principally associated with GO functions, including metabolic processes, immune system processes, and negative regulation of biological processes. The KEGG pathway revealed a significant enrichment of genes in Toll-like receptor cascades, ubiquitin-mediated proteolysis, and regulation of proteolysis (Fig. 4B). Additionally, the top 25 genes related to RNF115 were extracted from the Pathway Commons database to establish a PPI network. As displayed in Fig. 5A, 3 genes can regulate RNF115 expression (red lines), 18 genes can be regulated by RNF115 (blue lines), and 4 genes are other regulators with RNA115 (gray lines). Genes regulated by RNF115 were subjected to cBioPortal for correlation analysis with RNF115 in THCA based on the TCGA database. We found that RNF115 exerts positive regulation on APP, EGFR, FBXW11, HERC2, MYC, and UBE2W, and negatively regulates CDK10, ELAVL1, FOLR3, SFN, and TRADD (Fig. 5B). Subsequently, the genes above underwent analysis via GEPIA to evaluate their expression in THCA based on the TCGA database. The results showed that CDK10 and MYC expression was downregulated, while SFN expression was upregulated in THCA than in normal (*p* < 0.01; Fig. 5C).

**Fig. 6:**
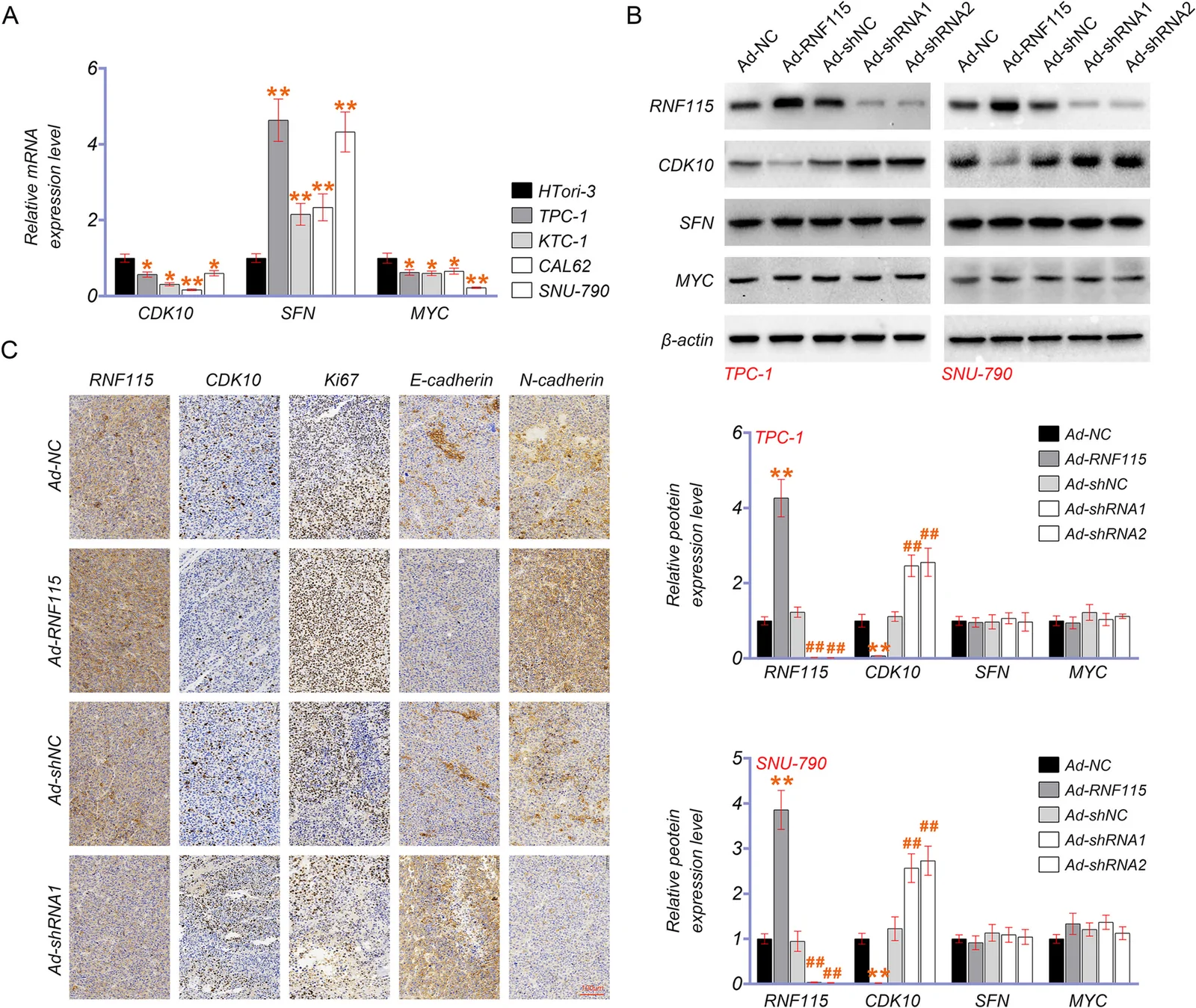
RNF115 downregulates the CDK10 expression in thyroid carcinoma (THCA) cells and tissues. **A** The expression of CDK10, SFN, and MYC in normal (HTori-3) and THCA cells (TPC-1, KTC-1, CAL62, and SNU-790) by RT-qPCR. *p < 0.05 and **p < 0.01 *vs.* HTori-3. **B** The CDK10, SFN, and MYC expression in TPC-1 and SNU-790 cells by western blotting. TPC-1 and SNU-790 cells were transfected with RNF115 overexpression (Ad-RNF115), knockdown (Ad-shRNA1 and Ad-shRNA2), or their negative controls (Ad-NC and Ad-shNC). **p < 0.01 *vs.* Ad-NC; ##p < 0.01 vs. Ad-shNC. **C** The expression of RNF115, CDK10, Ki67, E-cadherin, and N-cadherin in tumor tissues. BALB/c mice were injected with stably transfected TPC-1 cells into the lateral tail vein. After 30 days, mice were sacrificed to collect tumor tissues (scale bar = 200 µm)

Additionally, the expression levels of CDK10, SFN, and MYC were examined in human thyroid epithelial cells (HTori-3) as well as in THCA cell lines, including TPC-1, KTC-1, CAL62, and SNU-790. As anticipated, CDK10 and MYC had the lower expression, while SFN had higher expression in THCA cells than in normal HTori-3 cells (p < 0.05; Fig. 6A). estern blot analysis revealed that RNF115 overexpression led to a notable decrease in CDK10 protein levels in TPC-1 and SNU-790 cells, while RNF115 knockdown resulted in its upregulation (*p* < 0.01). Conversely, the expression levels of SFN and MYC remained largely unaffected (Fig. 6B). The suppressive influence of RNF115 on CDK10 expression was further corroborated in THCA tumor tissues through IHC. Meanwhile, RNF115 overexpression promotes the levels of Ki67 (a cell proliferation marker) and N-cadherin, as well as inhibiting E-cadherin expression in tumor tissues (Fig. 6C).

### RNF115 reduced the stability of CDK10 in THCA cells

**Fig. 7:**
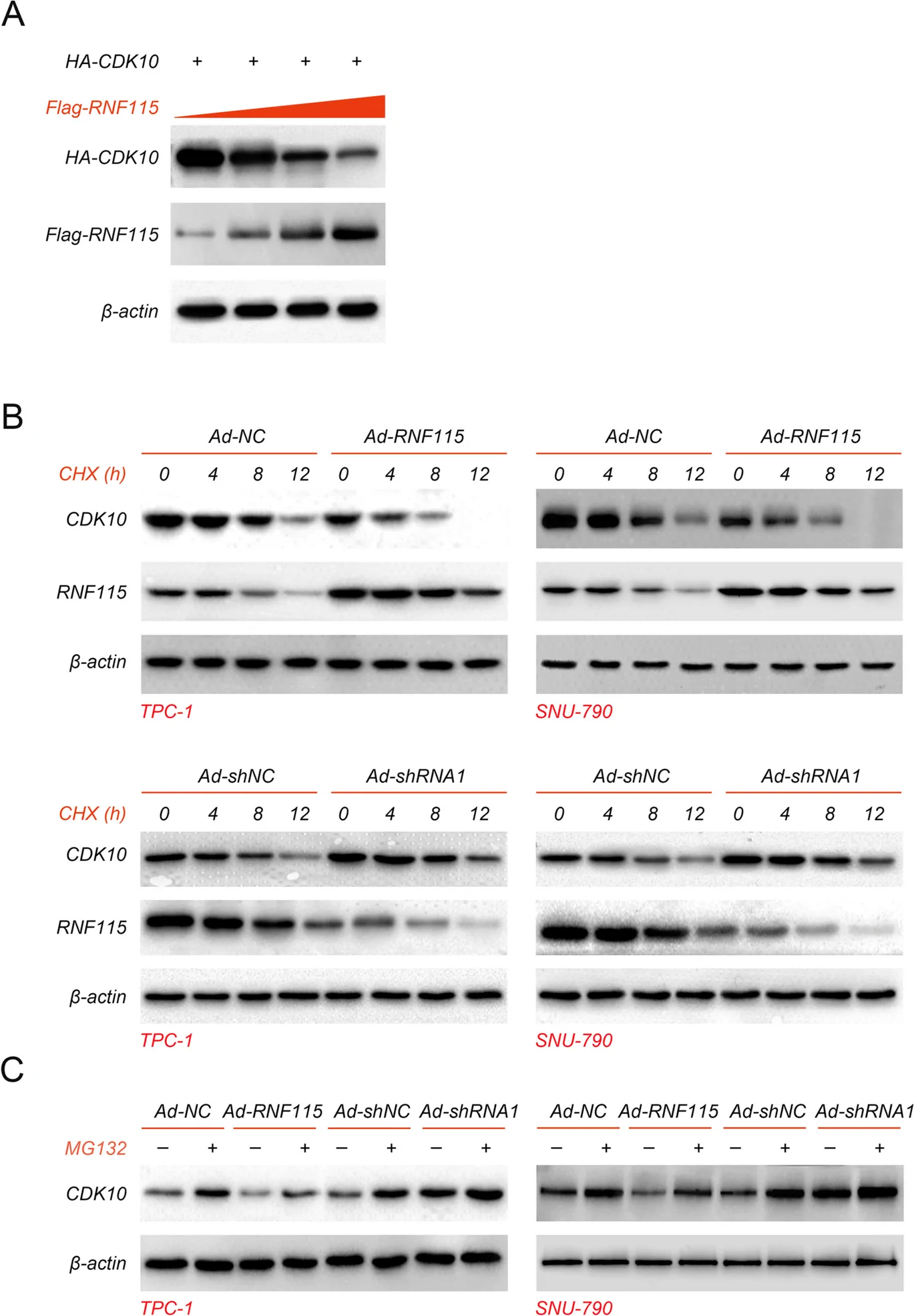
RNF115 reduces the stability of CDK10 in thyroid carcinoma (THCA) cells. **A** Co-immunoprecipitation identified the interaction between RNF115 and CDK10. **B-C** The expression of RNF115 and CDK10 in TPC-1 and SNU-790 cells by western blotting. Transfected cells were treated with CHX (a protein synthesis inhibitor) or MG132 (a proteasome inhibitor)

We further confirmed the regulatory effects of RNF115 on CDK10 in THCA cells. As depicted in Fig. 7A, the increased dosage of Flag-RNF115 led to a reduction in the protein levels of HK-CDK10. In addition, CHX (a protein synthesis inhibitor) was used to treat RNF115-overexpressed or -knocked down THCA cells for 0, 4, 8, and 12 h to explore the degradation effect of RNF115 on CDK10. Results showed that RNF115 overexpression accelerated the degradation of CDK10, while knockdown reduced the degradation in TPC-1 and SNU-790 cells with CHX administration (Fig. 7B). In contrast, the diminished CDK10 levels triggered by RNF115 overexpression were restored upon treatment with MG132 (a proteasome inhibitor) (Fig. 7C).

### RNF115 facilitated the ubiquitinated degradation of CDK10 and then activated the Raf-1 pathway in THCA cells

**Fig. 8:**
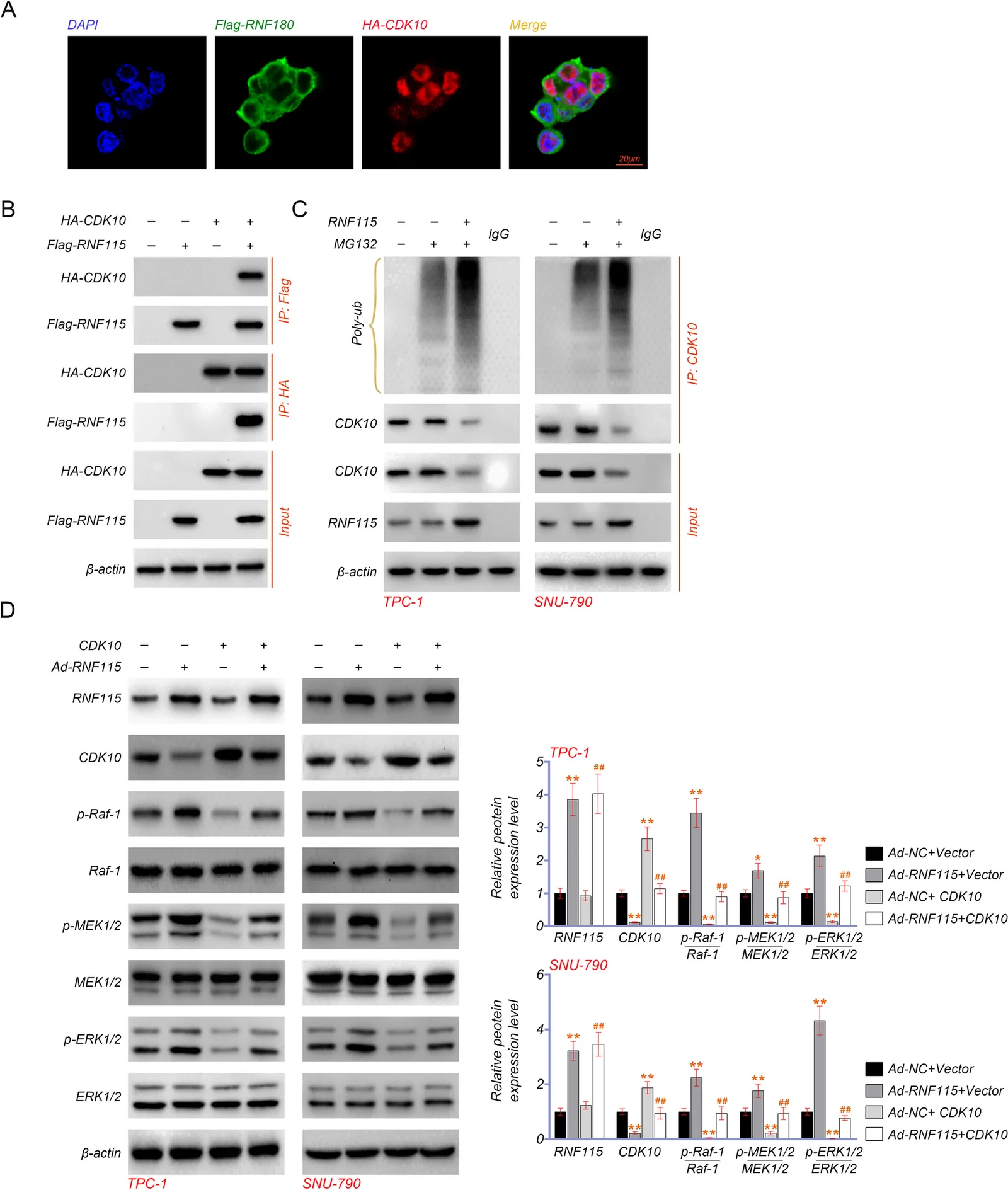
RNF115 facilitates the ubiquitinated degradation of CDK10 and then activates the Raf-1 pathway in thyroid carcinoma (THCA) cells. **A** Subcellular localization of RNF115 and CDK10 by immunofluorescent staining (scale bar = 20 µm). **B** The interaction between RNF115 and CDK10 by CoIP assay. **C** CDK10 ubiquitination was measured by western blotting. **D** Western blotting shows the expression of RNF115, CDK10, and Raf-1 pathway-related proteins in TPC-1 and SNU-790 cells. Cells were transfected with Ad-RNF115 and/or CDK10. *p < 0.05 and **p < 0.01 *vs.* Ad-NC + Vector; ##p < 0.01 vs. Ad-RNF115 + Vector

Additionally, using immunofluorescence staining, we discerned the subcellular locations of RNF115 and CDK10. The analysis revealed that Flag-RNF115 and HA-CDK10 predominantly shared a nuclear co-localization (Fig. 8A). The CoIP assay verified the interaction between RNF115 and CDK10 (Fig. 8B). Moreover, we found that RNF115 overexpression elevated CDK10 ubiquitination in TPC-1 and SNU-790 cells with MG132 treatment (Fig. 8C). Additionally, the Raf-1 signaling pathway is a critical signal node during ubiquitination, which determines the biological processes of tumor cells (Fan et al. 2020). Western blot analysis revealed that when RNF115 was overexpressed, there was a notable increase in the expression levels of p-Raf-1, p-MEK1/2, and p-ERK1/2. Conversely, an elevation in CDK10 levels led to a marked decrease in proteins related to the Raf-1 pathway (*p* < 0.05). CDK10 addition had a reversing effect on the promotive impact of RNF115 on the Raf-1 pathway in both TPC-1 and SNU-790 cells (p < 0.01; Fig. 8D).

### RNF115 promoted THCA cell proliferation and invasion by downregulating CDK10

**Fig. 9:**
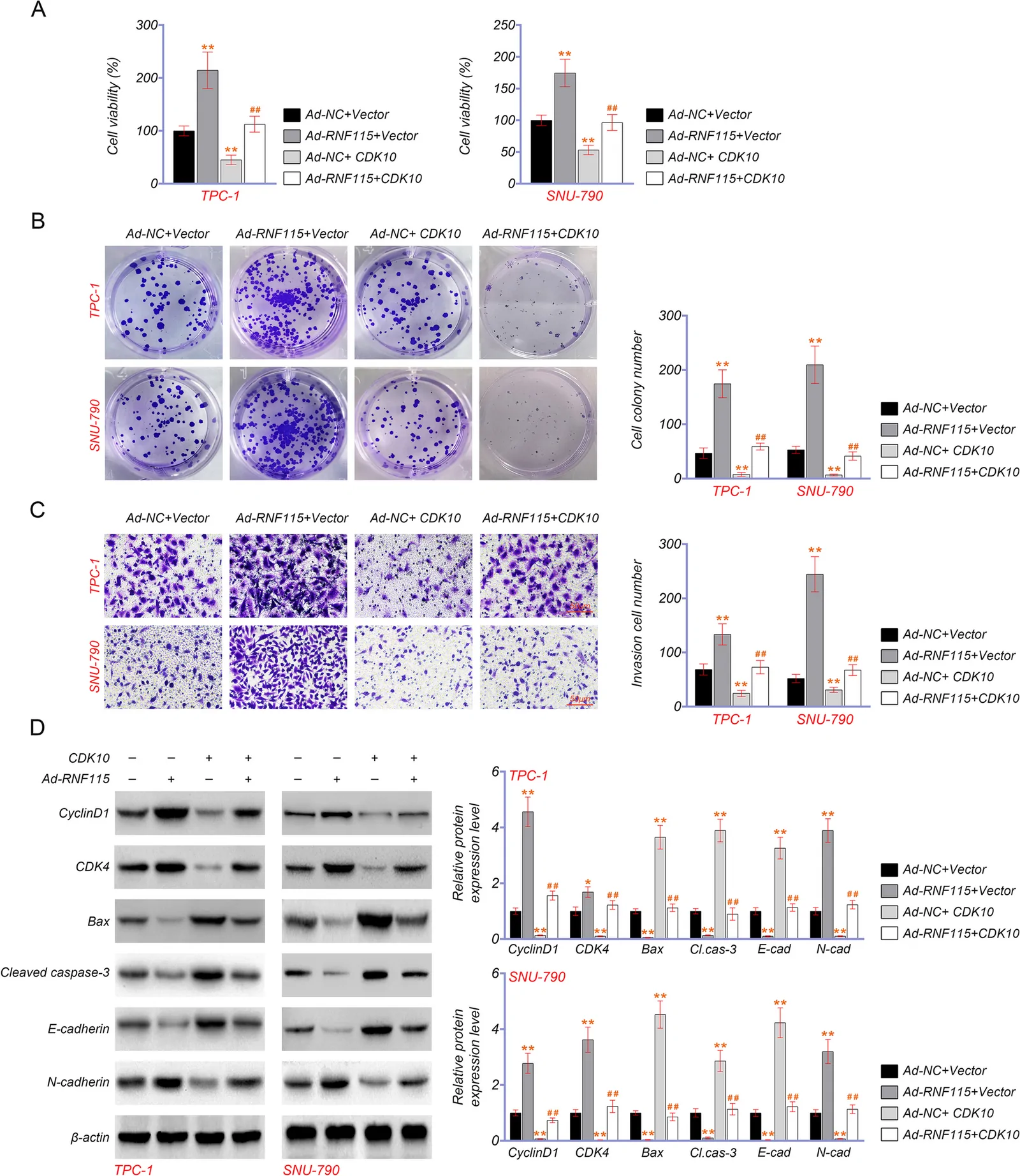
RNF115 promotes thyroid carcinoma (THCA) cell proliferation and invasion by downregulating CDK10. **A** Cell viability by MTT assay. **B** Cell proliferation by colony formation assay. **C** Cell invasion by Transwell assay (scale bar = 50 µm). **C** The expression of CyclinD1, CDK4, Bax, cleaved caspase-3, E-cadherin, and N-cadherin in TPC-1 and SNU-790 cells by western blotting. TPC-1 and SNU-790 cells were transfected with Ad-RNF115 and/or CDK10. *p < 0.05 and **p < 0.01 *vs.* Ad-NC + Vector; #p < 0.05 and ##p < 0.01 vs. Ad-RNF115 + Vector

We further confirmed the regulatory function of the RNF115/CDK10 axis in THCA cells. In contrast to RNF115 overexpression, CDK10 elevation reduced cell proliferation, migration, and invasion (p < 0.01; Figure S1B & 9A-C). Moreover, elevating CDK10 levels led to diminished expression of cell cycle-associated proteins (CyclinD1 and CDK4) and N-cadherin (p < 0.01). On the flip side, levels of Bax, Cleaved caspase-3, and E-cadherin saw an increase in both TPC-1 and SNU-790 cells (*p* < 0.01; Fig. 9D). Noteworthily, CDK10 addition offset the oncogenic effect of RNF115 on THCA (p < 0.05; Fig. 9A-D).

## Discussion

THCA is characterized as a malignancy noted for its aggressive disposition and an unfavorable prognosis (Araque et al. 2020). In this study, a significant upregulation of RNF115 was observed in both THCA tissues and cells. The significant enhancement of cell proliferation and tumor growth in THCA is observed upon the upregulation of RNF115. In particular, RNF115 overexpression facilitated the cell EMT *in vitro* and lung metastasis *in vivo* of THCA. Moreover, we further demonstrated that the promotive effect of RNF115 on THCA was achieved by ubiquitylating CDK10 and activating the Raf-1 pathway. Consequently, RNF115 functions as a promotor of tumorigenesis in THCA.

RNF115, known as an E3 ubiquitin ligase, is engaged in the swift turnover in the ubiquitin–proteasome pathway and participates in innate immunity (Zhang et al. 2020b; Li et al. 2020). Recent studies highlight its role in tumorigenesis and disease progression. RNF115 orchestrates the ubiquitination of specific proteins, influencing related signaling cascades. This modulation impacts viral infections, autoimmunity, cell growth, and tumor development (Wang et al. 2022). RNF115 has been identified as a regulator in lung adenocarcinoma, where it promotes cellular growth and metabolism while suppressing apoptosis. This is achieved through the ubiquitination of APC and p53 (Wu et al. 2021; Luo et al. 2020). Lu et al. indicated that RNF115 is overexpressed in human breast cancer and implicated in tumor pathogenesis and progression (Lu et al. 2019). Zhang et al. demonstrated that RNF115 acts on cell proliferation and DNA damage repair, a potential diagnostic biomarker and cancer therapeutic target (Zhang et al. 2020a). The findings indicate that RNF115 expression is increased in THCA tissues and cells, implying its potential as a prognostic biomarker for THCA. Functionally, RNF115 was shown to induce THCA cell proliferation and tumor growth. Meanwhile, RNF115 overexpression promotes THCA cell EMT and lung metastasis. Our findings offer convincing proof of RNF115 in the tumorigenesis and progression of THCA. Delving deeper into the regulatory roles of RNF115 in THCA is pivotal, as it could pave the way for the development of potent therapeutic interventions.

As an E3 ligase, RNF115 physically targets multiple proteins for ubiquitination and proteasomal degradation. Bioinformatics analyses unveiled the potential regulatory role of RNF115 over 18 proteins specific to THCA. A standout in this profile is the cyclin-dependent kinase 10 (CDK10), which manifests reduced expression in THCA as well as in gastric and biliary tract malignancies, corroborating previous studies (You et al. 2018; Yu et al. 2012). Of note, our data established a negative correlation between RNF115 elevation and CDK10 protein expression in THCA contexts. RNF115 overexpression also elevated CDK10 ubiquitination in THCA cells. The findings suggest that RNF115 may exert its oncogenic effect on THCA utilizing CDK10 degradation. Functionally, CDK10 overexpression reversed the promotive effect of RNF115 on THCA cell proliferation, invasion, and EMT. CDK10 is a CDC2-related serine/threonine kinase closely involved in cellular processes, including proliferation, transcription, and cell cycle regulation (Bazzi and Tai 2021). Moreover, our investigation revealed that RNF115 overexpression augmented the protein expression of CyclinD1 and CDK4 (positive regulators of cell cycle progression) in THCA cells. However, the CDK10 addition reversed their expression. This result suggests that RNF115 promotes the cancer cell cycle via CDK10 ubiquitination, thereby aggravating THCA progression.

**Fig. 10:**
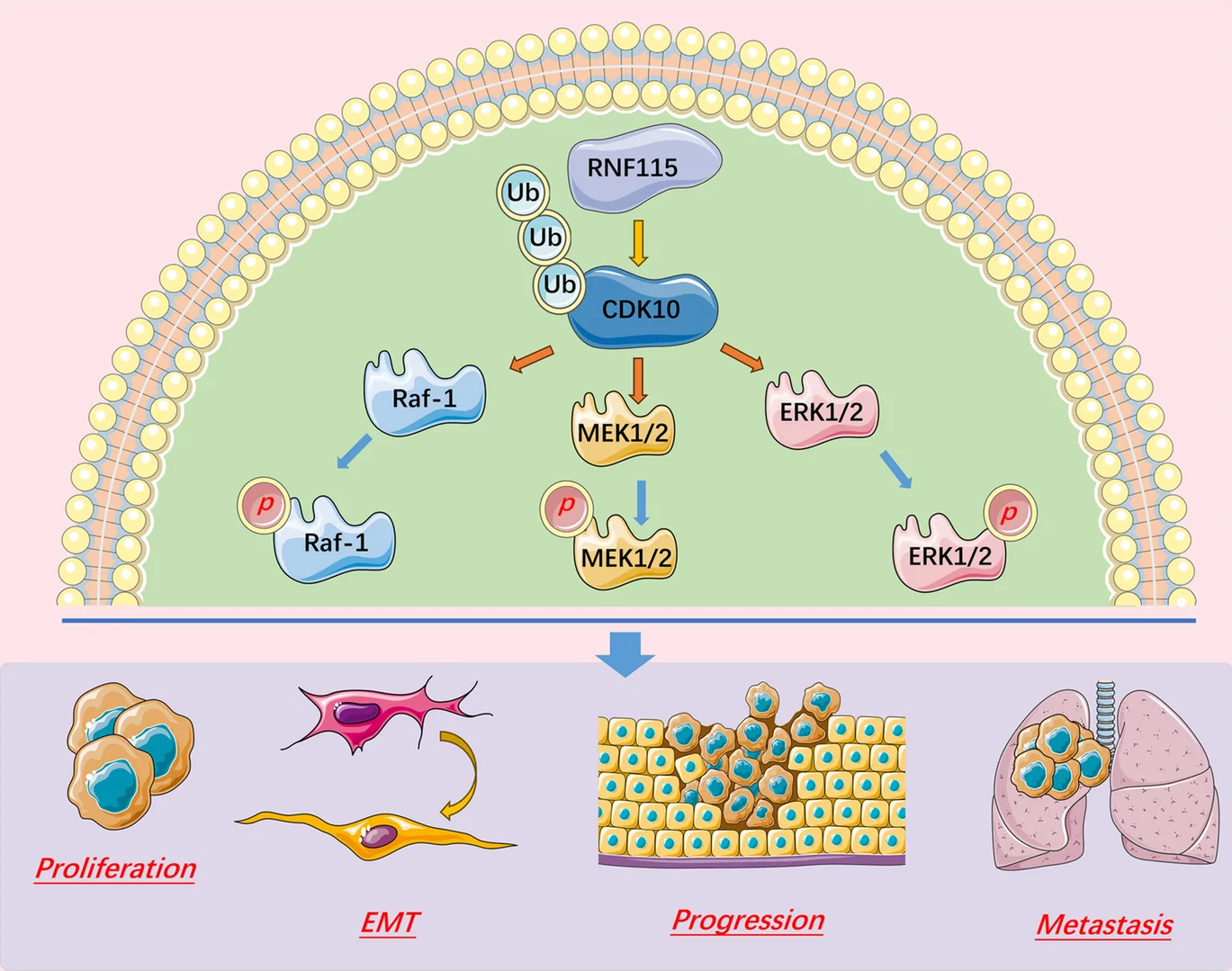
Graphical summary of effects and mechanisms of RNF115 on CDK10 degradation that regulate THCA progression

In conclusion, this study elucidates the role of RNF115 in promoting CDK10 ubiquitination and then activating Raf-1 signaling pathway, thereby accelerate THCA progression (Fig. 10). This sheds light on its regulatory function in the cell cycle. We provide a potential diagnostic and prognostic indicator for THCA, and corresponding mechanism investigation contributes to developing therapeutic strategies.

## Supplementary Information

Below is the link to the electronic supplementary material.

Supplementary file1 (TIF 6203 KB)

Supplementary file2 (DOCX 14 KB)

## Data Availability

The authors declare that all data supporting the findings of this study are available within the paper, and any raw data can be obtained from the corresponding author upon request.
