# Correction to: RNF115 aggravates tumor progression through regulation of CDK10 degradation in thyroid carcinoma

**DOI:** 10.1007/s10565-026-10253-5

**Published:** 2026-09-07

**Authors:** Jinxiang Zhu, Longwei Guo, Hao Dai, Zhiwei Zheng, Jinfeng Yan, Junsong Liu, Shaoqiang Zhang, Xiang Li, Xin Sun, Qian Zhao, Chongwen Xu

**Affiliations:** 1 Department of Otorhinolaryngology‑Head and Neck Surgery, the First Affiliated Hospital of Xi’an Jiaotong University, Western Yanta Road, Xi’an City, 710061 Shaanxi Province China; 2 Department of General Surgery, Shaanxi Provincial Cancer Hospital, Xi’an City, 710061 Shaanxi Province China; 3 Department of Radiation Oncology, the First Affiliated Hospital of Xi’an Jiaotong University, Xi’an City, 710061 Shaanxi Province China; 4 Department of The Third Ward of General Surgery, Rizhao People’s Hospital, Rizhao City, 276800 Shandong Province China; 5 Department of Thoracic Surgery, the First Affiliated Hospital of Xi’an Jiaotong University, Xi’an City, 710061 Shaanxi Province China

**Correction to: Cell Biol Toxicol (2024) 40:14**

**https://doi.org/10.1007/s10565-024-09845-w**

Following publication of the original article, readers brought several figure discrepancies to the authors' and Editor’s attention. Specifically, two transwell assay images in Figure 3A were misused, and the dimensions of the excised tumors in the left panel of Figure 2D did not correlate with the reported volume sizes in the right panel. Upon re-examining the original data files, the authors identified additional assembly and placement errors, including the cell colony images in Figures 2C and 9B, the Raf-1 immunoblotting band for SUN-790 in Figure 8D, and the transwell assay images in Figure 9C. These discrepancies resulted solely from file management errors during manuscript preparation. To rectify these issues, the authors repeated the relevant experiments, recalculated the tumor volumes, and performed an unpaired two-tailed t-test for statistical analysis in Figure 2D. The corrected figures are provided in this Corrigendum. The authors confirm that these corrections do not alter the scientific findings or overall conclusions of the paper. The authors sincerely apologize for these errors and any inconvenience caused to the readership.

The updated Figures 2, 3, 8 and 9 are shown below.

**Fig. 2:**
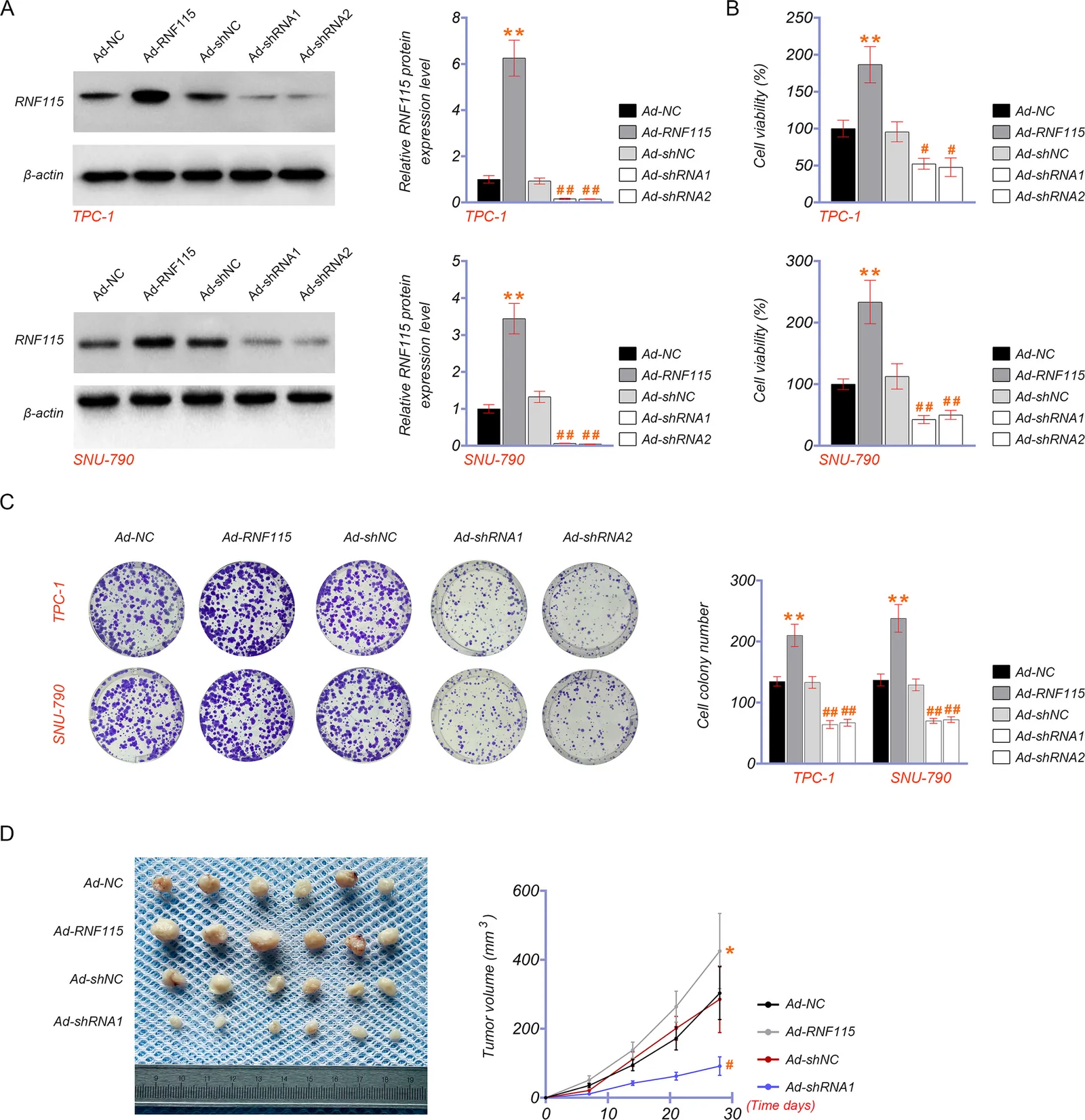
RNF115 promotes cell proliferation and tumor growth of thyroid carcinoma (THCA). **A** The protein expression of RNF115 in TPC-1 and SNU-790 cells. **B** Cell viability by MTT assay. **C** Cell proliferation by colony formation assay. **A-C** TPC-1 and SNU-790 cells were transfected with RNF115 overexpression (Ad-RNF115), knockdown (Ad-shRNA1 and Ad-shRNA2), or their negative controls (Ad-NC and Ad-shNC). **D** Xenograft Tumor volume. BALB/c mice were injected with stably transfected TPC-1 cells into the lateral tail vein. Tumor volume was recorded every seven days. *p < 0.05 and **p < 0.01 vs. Ad-NC; #p < 0.05 and ##p < 0.01 vs. AdshNC

**Fig. 3:**
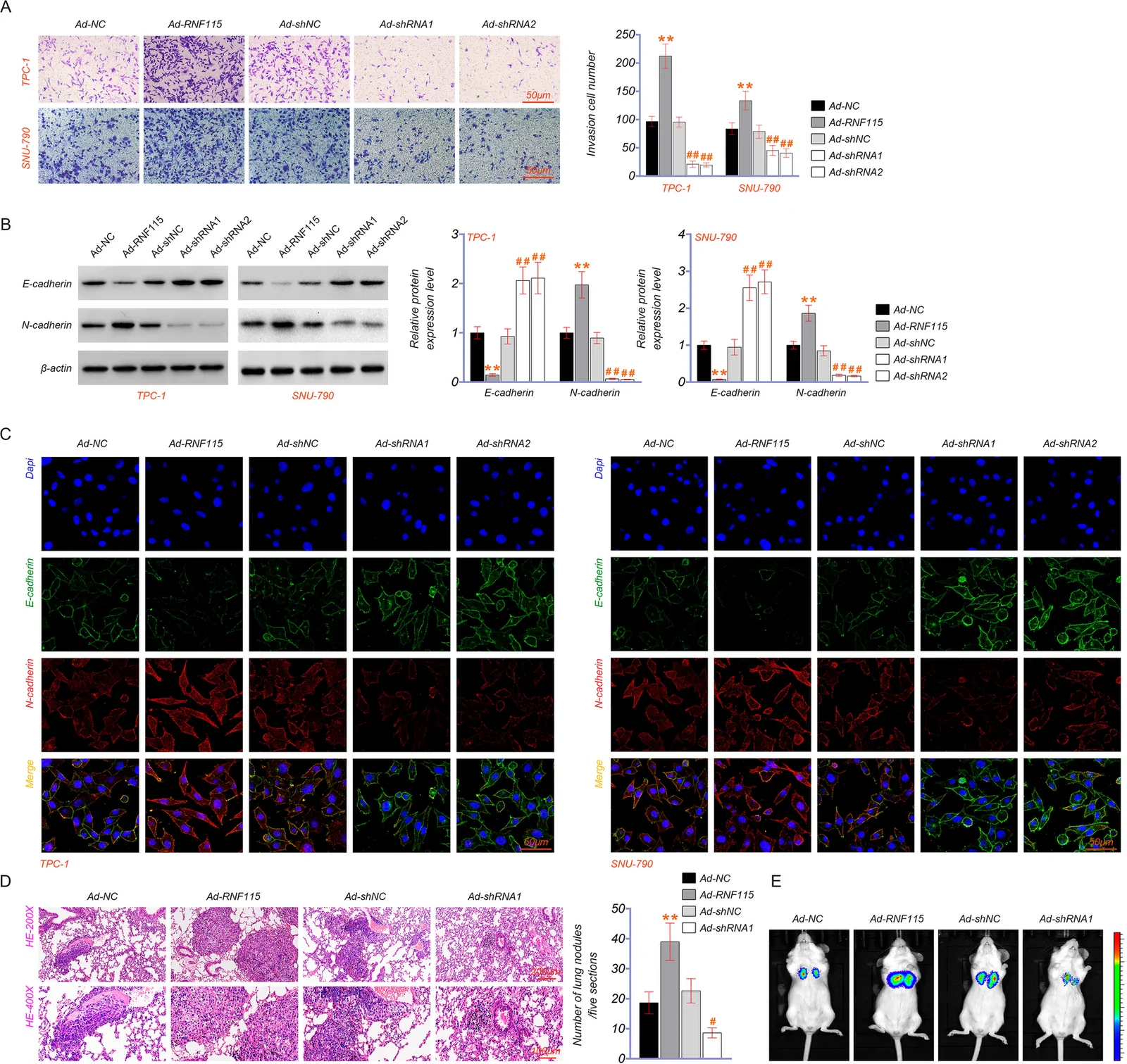
RNF115 promotes cell epithelial-mesenchymal transition (EMT) and tumor lung metastasis of thyroid carcinoma (THCA). **A** Cell invasion by Trans well assay (scale bar = 50 μm). **B-C** EMT-related proteins (E-cadherin and N-cadherin) were expressed in cells by western blotting and immunofluorescence (scale bar = 50 μm). **A-C** TPC-1 and SNU-790 cells were transfected with RNF115 overexpression (Ad-RNF115), knockdown (Ad-shRNA1 and Ad-shRNA2), or their negative controls (Ad-NC and Ad-shNC). **D-E** HE staining (200 × and 400 × magnifications) and imaging measured lung metastasis of the THCA tumor. BALB/c mice were injected with stably transfected TPC-1 cells into the lateral tail vein. Tumor volume was recorded every seven days. **p < 0.01 vs. Ad-NC; #p < 0.05 and ##p < 0.01 vs. Ad-shNC

**Fig. 8:**
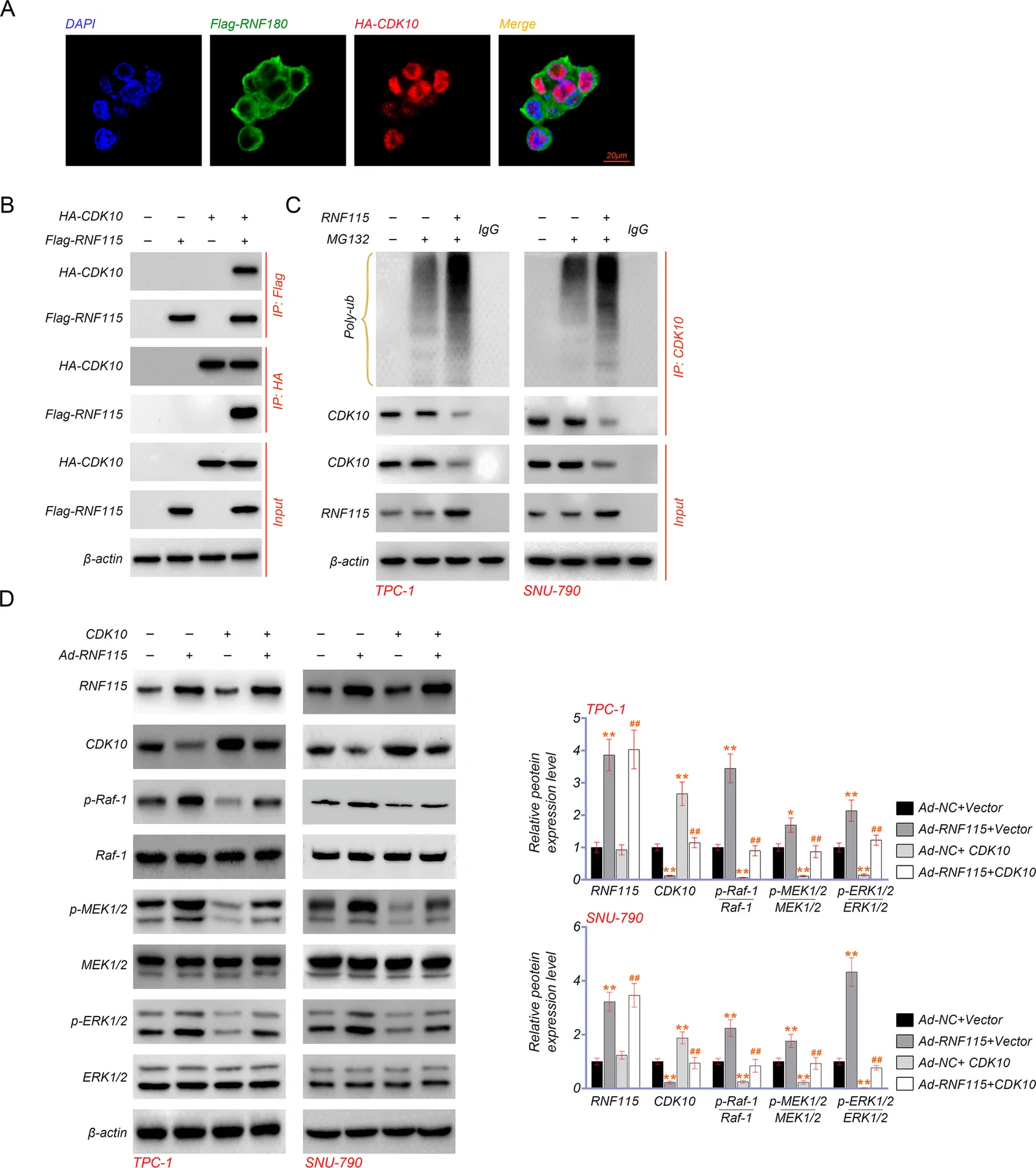
RNF115 facilitates the ubiquitinated degradation of CDK10 and then activates the Raf-1 pathway in thyroid carcinoma (THCA) cells. **A** Subcellular localization of RNF115 and CDK10 by immunofluorescent staining (scale bar = 20 μm). **B** The interaction between RNF115 and CDK10 by CoIP assay. **C** CDK10 ubiquitination was measured by western blotting. **D** Western blotting shows the expression of RNF115, CDK10, and Raf-1 pathway-related proteins in TPC-1 and SNU-790 cells. Cells were transfected with Ad-RNF115 and/or CDK10. *p < 0.05 and **p < 0.01 vs. Ad-NC + Vector; ##p < 0.01 vs. Ad-RNF115 + Vector

**Fig. 9:**
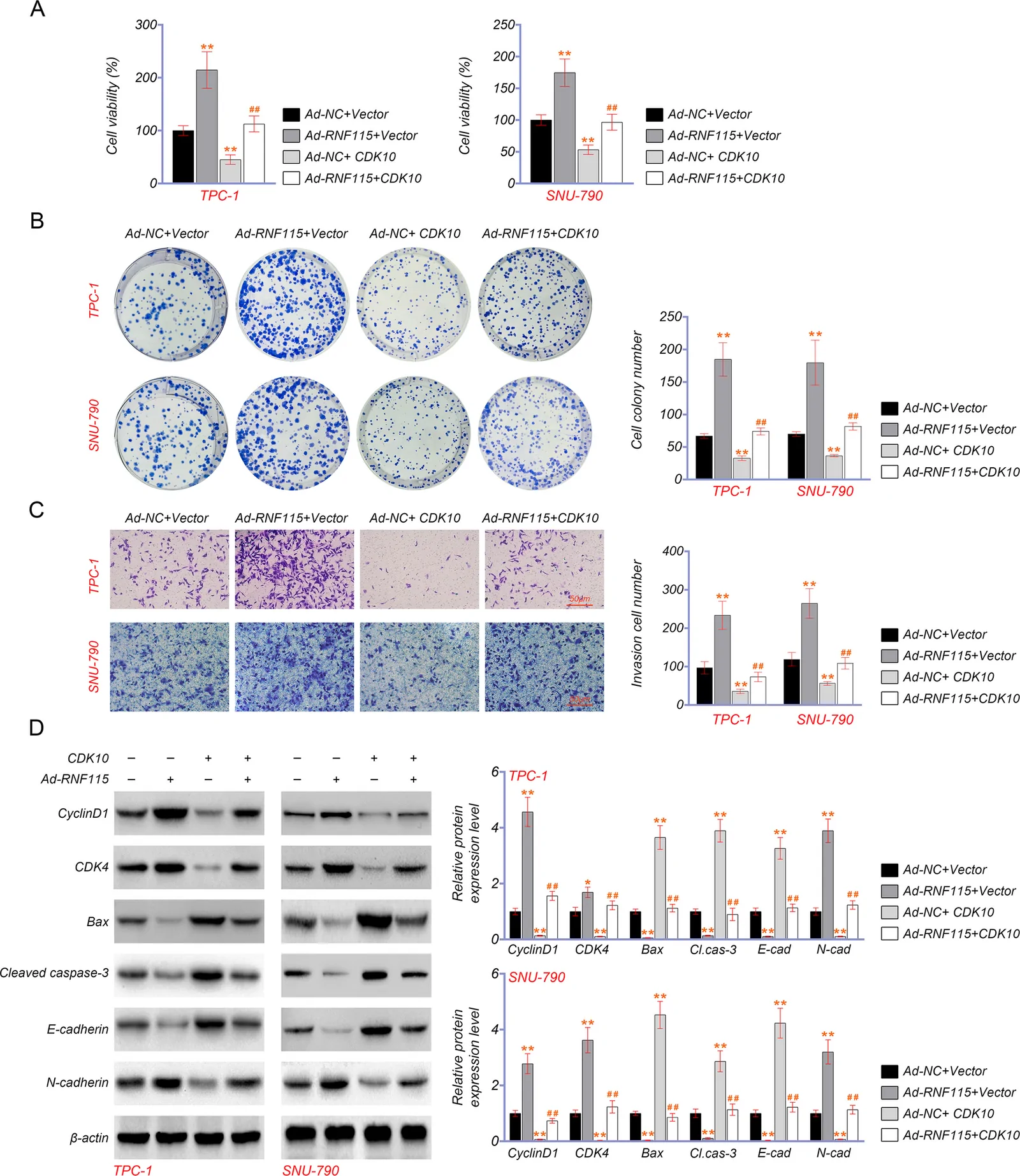
RNF115 promotes thyroid carcinoma (THCA) cell proliferation and invasion by downregulating CDK10. **A** Cell viability by MTT assay. **B** Cell proliferation by colony formation assay. **C** Cell invasion by Transwell assay (scale bar = 50 μm). **C** The expression of CyclinD1, CDK4, Bax, cleaved caspase-3, E-cadherin, and N-cadherin in TPC-1 and SNU-790 cells by western blotting. TPC-1 and SNU-790 cells were transfected with Ad-RNF115 and/or CDK10. *p < 0.05 and **p < 0.01 vs. Ad-NC + Vector; #p < 0.05 and ##p < 0.01 vs. Ad-RNF115 + Vector

