# Supplementary figures and images for: RNF115 aggravates tumor progression through regulation of CDK10 degradation in thyroid carcinoma
Source: Cell Biol Toxicol. 2024 Feb 20;40(1):14. doi: 10.1007/s10565-024-09845-w (PMC10879231; doi:10.1007/s10565-024-09845-w)

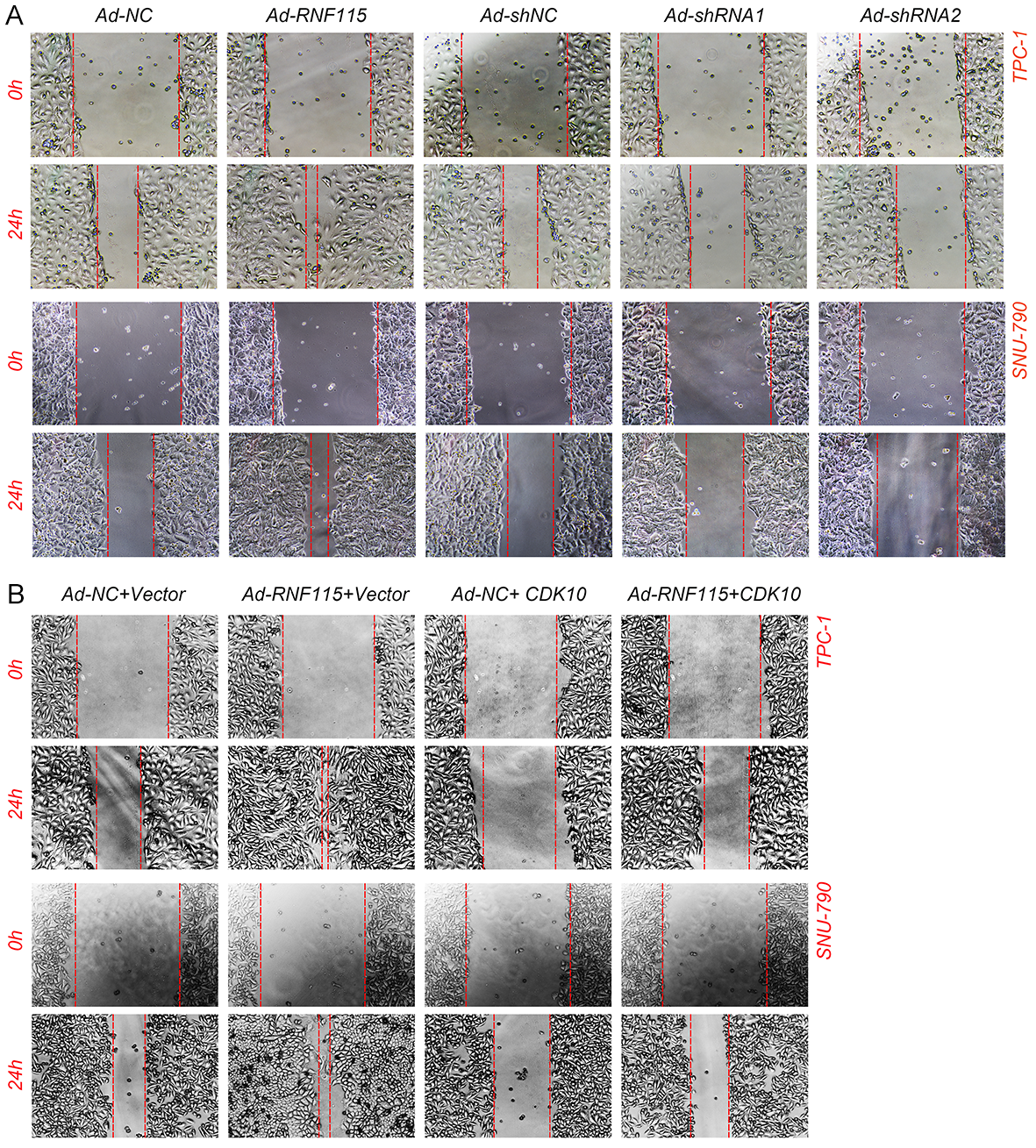

Supplement: Supplementary file 1 — Supplementary file1 (TIF 6203 KB) [file 10565_2024_9845_MOESM1_ESM.tif]
