# Supplementary material for: RNF115 aggravates tumor progression through regulation of CDK10 degradation in thyroid carcinoma
Source: Cell Biol Toxicol. 2024 Feb 20;40(1):14. doi: 10.1007/s10565-024-09845-w (PMC10879231; doi:10.1007/s10565-024-09845-w)
Supplement: Supplementary file 2 — Supplementary file2 (DOCX 14 KB) [file 10565_2024_9845_MOESM2_ESM.docx]

**Table S1.** Primer sequences for RT-qPCR.

| Gene | Primer sequence (5’-3’) |
| --- | --- |
| RNF115 | Forward: AGCTGACAAGGAAAAGATCACA |
|  | Reverse: CTAGCCACGGCACAATACAAC |
| CDK10 | Forward: GACCTGAAGGTTTCCAAC |
|  | Reverse: ACATGTCGATGCTGGTGG |
| SFN | Forward: AGCCCTTTGGAGCAAGAACAG |
|  | Reverse: ACAACCTGACACTGTGGACG |
| MYC | Forward: CCTCCACTCGGAAGGACTATC |
|  | Reverse: TGTTCGCCTCTTGACATTCTC |
| β-actin | Forward: ACGGCCAGGTCATCACTATT |
|  | Reverse: TGGCATAGAGGTCTTTACGGA |
